# Array Comparative Genomic Hybridization Analysis Reveals Significantly Enriched Pathways in Canine Oral Melanoma

**DOI:** 10.3389/fonc.2019.01397

**Published:** 2019-12-12

**Authors:** Ginevra Brocca, Serena Ferraresso, Clarissa Zamboni, Elena M. Martinez-Merlo, Silvia Ferro, Michael H. Goldschmidt, Massimo Castagnaro

**Affiliations:** ^1^Department of Comparative Biomedicine and Food Science, University of Padua, Legnaro, Italy; ^2^Department of Animal Medicine and Surgery, Complutense University, Madrid, Spain; ^3^School of Veterinary Medicine, University of Pennsylvania, Philadelphia, PA, United States

**Keywords:** angiogenesis, array comparative genomic hybridization, canine oral melanoma, comparative oncology, copy number aberrations, mucosal melanoma, pathway enrichment analysis

## Abstract

Human Mucosal Melanoma (hMM) is an aggressive neoplasm of neuroectodermal origin with distinctive features from the more common cutaneous form of malignant melanoma (cMM). At the molecular level, hMMs are characterized by large chromosomal aberrations rather than single-nucleotide mutations, typically observed in cMM. Given the scarcity of available cases, there have been many attempts to establish a reliable animal model. In pet dogs, Canine Oral Melanoma (COM) is the most common malignant tumor of the oral cavity, sharing clinical and histological aspects with hMM. To improve the knowledge about COM's genomic DNA alterations, in the present work, formalin-fixed, paraffin-embedded (FFPE) samples of COM from different European archives were collected to set up an array Comparative Genomic Hybridization (aCGH) analysis to estimate recurrent Copy Number Aberrations (CNAs). DNA was extracted in parallel from tumor and healthy fractions and 19 specimens were successfully submitted to labeling and competitive hybridization. Data were statistically analyzed through GISTIC2.0 and a pathway-enrichment analysis was performed with ClueGO. Recurrent gained regions were detected, affecting chromosomes CFA 10, 13 and 30, while lost regions involved chromosomes CFA 10, 11, 22, and 30. In particular, CFA 13 showed a whole-chromosome gain in 37% of the samples, while CFA 22 showed a whole-chromosome loss in 25%. A distinctive sigmoidal trend was observed in CFA 10 and 30 in 25 and 30% of the samples, respectively. Comparative analysis revealed that COM and hMM share common chromosomal changes in 32 regions. MAPK- and PI3K-related genes were the most frequently involved, while pathway analysis revealed statistically significant perturbation of cancer-related biological processes such as immune response, drug metabolism, melanocytes homeostasis, and neo-angiogenesis. The latter is a new evidence of a significant involvement of neovascularization-related pathways in COMs and can provide the rationale for future application in anti-cancer targeted therapies.

## Introduction

Human melanomas of mucosal sites (human Mucosal Melanoma, hMM) are neoplastic diseases of neuroectodermal origin, arising from non-cutaneous melanocytes migrated from the neural crest during embryogenesis (1–4). Although still not fully characterized, hMMs show to rely on numerous copy number changes and whole chromosomes gains or losses, rather than on single-nucleotide mutations, and they lack the typical UV-signature of the cutaneous malignant melanomas (cMM) (4–8). Large chromosomal aberrations, known to be deeply involved in solid tumors development (9), were investigated in hMMs through numerous techniques. Up to date, promising recurrent regions of gains and losses were identified (5) and confirmed by several investigations (4, 7, 8), in particular amplified portions of HSA 12q and 5p, which encode for genes as CDK4 and TERT, respectively (7). In addition, CCND1, KIT, and VEGFRA were proposed by a recent review (10) as targets for future investigations. hMMs represent only the 1.3% of all reported melanomas (1) and they may arise from different sites, as head-and-neck, female genital tract, and anal/rectal mucosa, with a respective 5 years survival rate of 31.7, 11.4, and 19.8%, while cMM has a 5 years survival rate of 80.8% (1). The highly aggressive biological behavior of hMMs (11) and the scarcity of available cases led to many attempts to establish a reliable animal model for the study of this life-threatening disease. Various *in vivo* models have been proposed for melanocytic derived-tumors through genetically engineered mice and zebrafish (12). Relevant limitations of these models are the lack of tumor population heterogeneity, combined with the longtime of tumor formation (12, 13). Altogether, these studies revealed the necessity of a spontaneous tumor model in non-engineered animals. Among companion animals, equine's primary melanomas have been taken into consideration as a model for hMMs' aberrations (8); however, they showed to have fewer copy number changes compared to hMM, making them a non-fitting model. On the basis of their greater genetic proximity with humans than other models proposed, dogs appear to be a more adequate preclinical surrogate (14). Canine tumors arise spontaneously in an intact immune system, often at a higher rate than in humans, and pet dogs share the same environmental risk factors with the owners. Moreover, dogs have a shorter lifespan and a more rapid neoplastic disease course (15, 16). Canine Oral Melanomas (COMs), the most common malignant tumor of the canine oral cavity (2, 17, 18), are characterized by a clinical evolution and progression, a tendency for local invasion and metastasis (2, 19–22), and a resistance to chemotherapy and radiation therapy (15, 20, 23), similar to hMM. In 2012, the National Cancer Institute Comparative Melanoma Tumor Board compared histological features of COM and canine melanomas arising in other sites (skin and acral) with hMM and cMM, finding a complete concordance between COMs and hMMs, and suggesting a common enrichment of PI3K and MAPK pathways (13). Given these promising results, the Board strongly encouraged validation of COM as a clinical model for hMM, by deepening the correlation of possible chromosomal, epigenetic and transcriptomic alterations. Molecular studies on COMs detected recurrent gains in CFA 13 and 17, and recurrent losses in CFA 2 and 22 (8, 24). A distinctive sigmoidal trend was also highlighted in CFA 30, with the alternation of gained and lost regions (8, 24). Although a large variety of gained and deleted genes was detected, some studies revealed discordant results indicating the need for further investigation on COMs' genetic landscape. In this work, DNA from formalin-fixed, paraffin-embedded (FFPE) samples of COM was collected from two European archives and analyzed through array Comparative Genomic Hybridization (aCGH). This technique takes advantage of the competitive hybridization of matched healthy and pathologic genomic DNA in parallel-extracted from FFPE samples, to estimate recurrent somatic Copy Number Aberrations (CNAs) characteristic of the cluster analyzed.

## Materials and Methods

### Samples Collection and Selection

FFPE samples were collected from the archives of the Universities of Padua and Madrid. Initial inclusion criteria for the collection of the samples were a certain diagnosis of COM and sufficient material for nucleic acid extraction. Once collected, one 4 μm-thick slide was cut from each block and stained with a routine hematoxylin-eosin (H&E) protocol for a second evaluation. To be included in the study, the H&E slides were reviewed independently by two board-certified veterinary pathologists (American and European) and one expert veterinary pathologist to unequivocally confirm the initial diagnosis of COM, and to assess the presence of an adequate amount of healthy tissue suitable for the nucleic acid extraction. Diagnostic criteria were based upon the guidelines of the World Health Organization (25) and amelanotic specimens were evaluated through anti-Melan-A and anti-PNL2 antibodies. Forty samples were finally evaluated as adequate.

### Nucleic Acid Extraction and Purification From FFPE Tissue

By using H&E stained slides as a guide, the paraffin blocks were incised in order to separate the tumor bulk from the healthy tissue. Sections 20 μm-thick were then cut from the blocks using a microtome with disposable blades. Tumor and healthy tissues were then scraped from the slides and put in two different 1.5 ml Eppendorf to be extracted separately. When necessary, more sections were cut in order to provide an adequate amount of healthy tissue material. Care was taken to avoid any possible contamination between tumor and healthy tissue and between different samples, by cleaning microtome, blades, and instruments after processing each specimen. Genomic DNA was extracted using the All-Prep DNA-RNA FFPE KIT (Qiagen^®^) according to the manufacturer's instructions, with the use of a heptane solution for deparaffinization steps. Quality and quantity of the extracted DNA were assessed via spectrophotometry with a Nanodrop ND-1000 (Life Technologies^®^), while its integrity was checked with an agarose gel electrophoresis, showing a marked degree of degradation in all samples. Only samples with a A^260^/A^230^ ratio of at least 1.5 and a yield of DNA of at least 450 ng (for both pathological and healthy sections) were admitted to the following steps.

### Array Comparative Genomic Hybridization

Genomic DNA from 24 samples was subjected to the cyanine labeling using the SureTag DNA Labeling Kit: DNA extracted from pathological and healthy fractions was labeled independently with Cy 3-deoxyuridine triphosphate (dUTP) and cyanine 5-dUTP, respectively. Cyanine incorporation and final concentration were calculated via spectrophotometry with a Nanodrop ND-1000 and the specific activity was calculated for each sample. Twenty samples, which reached an adequate matched tumor/healthy Cy3 and Cy5 specific activity, were then co-hybridized to a 180,000-feature SurePrint G3 Canine CGH Microarray (4–180 K, Agilent Technologies), comprising repeat-masked 60-mer oligonucleotides distributed at ~2.7 Kb intervals throughout the dog CanFam2 genome assembly. After 24 h of incubation at 65° and 20 rpm, arrays were washed following the manufacturer's instruction and scanned at 3 μm using an Agilent G2565CA scanner. Image data were processed using Feature Extraction version 11.5, and Genomic Workbench version 7.0.

### CNAs Analysis

Data were filtered to exclude probes exhibiting non-uniform hybridization or signal saturation and were normalized using the centralization algorithm with a threshold of eight and fuzzy ON. The ADM-2 algorithm was applied to define CNAs using a “three probes minimum” filter. Only autosomes were analyzed. The Cy5/Cy3 intensity ratios for each spot were converted into log2 ratios. Aberrant chromosome intervals were selected by using Agilent Genomic Workbench v. 7.0. A copy number gain was defined as a log2 ratio >0.25 and a copy number loss was defined as a log2 ratio < -0.25. Chromosomal locations were defined in terms of their Megabase (Mb) position. To identify significant CNAs the Genomic Identification of Significant Targets in Cancer (GISTIC2.0) (26) algorithm was also applied, as implemented in CGHtools software. The GISTIC2.0 module identifies regions of the genome that are significantly amplified or deleted across samples. Each aberration is assigned a G-score that considers the amplitude as well as the frequency of its occurrence across samples. False Discovery Rate q-values are then calculated for the aberrant regions, and regions with q-values below a user-defined threshold are considered significant. Log2ratios ≥0.25 and ≤ –0.25 were assigned as the threshold for gain and loss detection, while amplification and deletion were defined as having a log2 ratio ≥1 and ≤ –1. False Discovery Rate (FDR) ≤ 0.05 was set as the limit of significance.

### Comparison Between Canine and Human CNAs

To compare the canine CNA profile with aberrations already described in the recent human literature, orthologous regions were identified using the Liftover Batch Coordinate Conversion Tool (http://genome.ucsc.edu/cgi-bin/hgLiftOver), as already done in previous studies (8, 24). In summary, the genome coordinates of the 180.000 60-mer probes of each array were mapped firstly to the canine reference genome CanFam3.1, and then to the human reference genome GRCh38/hg38. The syntenic human regions were then compared with published data (5, 7, 8, 24), to detect those regions shared by both hMMs and COMs. A comparative analysis between data produced herein, and recently published studies regarding the detection of CNAs in the canine genome through several techniques (as WES, WGS, aCGH, and FISH) (8, 24, 27, 28), was also performed.

### Pathway Enrichment Analysis

Orthologous human genes were identified using the Ensemble Genome Browser (http://www.ensembl.org/index.html) and four lists of genes were employed for pathway analysis: (i) Gains with penetrance ≥25% (GR25), (ii) Gains with penetrance ≥40% (GR40), (iii) Losses with penetrance ≥25% (LR25) and (iv) regions highlighted as significant by Gistic analysis (GS). Genes were analyzed as human orthologs using the ClueGo plugin (29) for the software Cytoscape 3.7.1, an open-source Java tool that extracts the non-redundant biological information for large clusters of genes. In ClueGO, the kappa score is used to define term-term interrelations (edges), and functional groups based on shared genes between terms. Here, *Homo sapiens* was used as the control organism, and genes were uploaded as human orthologs named by the SymbolID. The genes were assigned to a network based on the updated ontologies: KEGG, GO Biological process, GO Immuno, REACTOME, and WIKIPATHWAYS. The significance of each term was calculated with a standard hypergeometric two-sided test. Networks were created on the basis of a kappa score threshold of 0.5 and a minimum of 3 genes in every network forming at least 10% of the total associated genes in each particular network, as previously done (28). Pathways' *P*-values were adjusted with Benjamini-Hochberg and the “fusion” option was also applied to reduce the redundancy. Pathways were then represented taking advantage of Cytoscape's complex visualization environment, as kappa score-based functional groups, and named by the most significant term of each group.

### Immunohistochemistry and Immunohistochemical Assessment

For each of the 20 samples analyzed through the aCGH technique, a 4 μm-thick section was cut and mounted on a polarized glass slide (Superfrost^®^ Plus, Thermo Scientific^®^), and tested with the mouse monoclonal antibody Ki67 (Dako^®^) diluted 1:50. Immunohistochemistry was performed with an automatic immunostainer (Ventana Benchmark GX, Roche-Diagnostic) using an ultraView universal alkaline phosphatase RED detection kit (Ventana Medical System Inc.), which provides a red chromogen reaction, and hematoxylin counterstain. The use of the red chromogen allowed avoiding bleaching reactions in pigmented COMs, in which DAB chromogen is often unusable, preserving the integrity of antigens. A Ki67 index was established for each sample on the base of the methodology described by Bergin et al. (30), which showed to be prognostic with a cutoff of 19.5 average cells per high power field (hpf).

### Data Access

The data discussed in this publication have been deposited in NCBI's Gene Expression Omnibus (31), and are accessible through GEO Series accession number GSE131923 (https://www.ncbi.nlm.nih.gov/geo/query/acc.cgi?acc=~GSE131923).

## Results

### Collected Samples and Immunohistochemical Analysis

A total of 20 samples, inclusive of the tumor and matched normal tissue, were selected for cyanine labeling and showed both an adequate yield and an adequate specific activity to be further subjected to the aCGH analysis. Only one case showed poor quality of hybridization and was excluded from this study, bringing the number of samples to 19. For each sample, an IHC with the Ki67 antibody was successfully performed, and the Ki67 index was established. Based on the study conducted by Bergin et al. (30), we established a threshold Ki67 value of >19.5 for the prediction of death (or euthanasia) due to melanoma by 1 year post-diagnosis. Based on the Ki67 value, samples were then classified as with a GOOD (G) or BAD (B) possible prognosis. The G group included 5/20 samples, and the B group 15/20 (including the one excluded from the aCGH cohort). Samples and available clinical data of dogs from which they were collected are listed in Table S1 in Supplementary Materials.

### Genomic Pattern of Aberration

**Figure 1:**
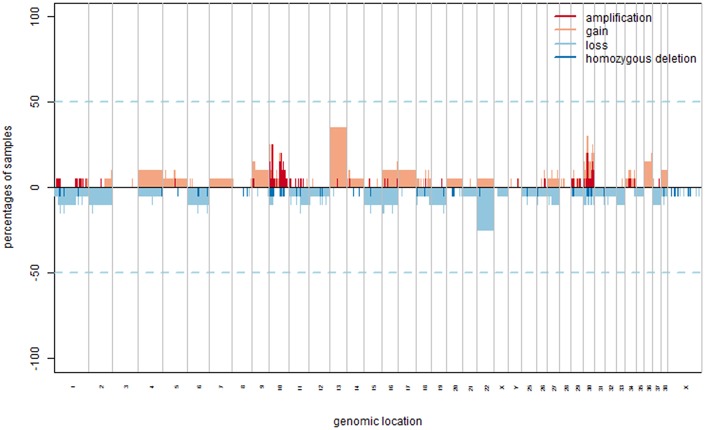
CNAs in COM. Copy number gains and amplification are indicated in orange and red, respectively representing a log_2_ ratio ≥0.25 and ≥1. Copy number losses and deletion are indicated in light and dark blue, respectively representing a log_2_ ratio ≤–0.25 and ≤–1.

CNA analysis allowed the identification of both focal and broad (near the size of a chromosome arm) chromosomal aberrations, distinguished in gains and losses (Figure 1). Two samples (A5, A35) did not present any aberration, while in the remaining ones, the mean number of aberrations per sample was 27.6 (range: 2–71). The pattern of genomic aberrations was evaluated for gained and lost regions with a penetrance ≥25% and consisted of 53 gained regions, with size ranging from 12.7 Kb to 30.9 Mb (with a mean length of 0.7 Mb), and 20 lost regions ranging from 60 bp to 40.5 Mb (mean length of 2 Mb).

The most frequently gained regions (penetrance ≥25%) affected chromosomes CFA 10, 13, and 30, while lost regions involved most frequently chromosomes CFA 10, 11, 22, and 30. Among the regions with gains, 8 showed a penetrance ≥40%, with regions chr30:17522685–17773010 and chr30:17847674–18058012 showing 45% penetrance. Among regions with losses, nine had a penetrance ≥30% and the most frequent loss was chr11:41248370–41248429, with a 35% penetrance. CNAs with penetrance ≥25% and corresponding genes are listed in Table S2 in Supplementary Materials. Chromosomes that appeared to be more affected by gains and losses were CFA 13 and 22. CFA 13 showed a whole-chromosome gain in the 37% (7/19) of the samples, while CFA 22 showed a whole-chromosome loss in the 25% (5/19) of the samples, with the loss of region 0.2 to 54 Mb that reached a 30% penetrance. Additionally, a recurrent and distinctive alternation of gained and lost regions (sigmoidal trend) was observed on CFA 10 (25% of the samples, 5/19) and 30 (30% of the samples, 6/19). No aberrations significantly associated with a Ki67 index greater or lower than 19.5 were identified. The most frequent aberration observed was the loss of region chr11:41248370–41248429 in the G group, recurrent in three out of five samples. Microarray data were then interrogated using the GISTIC2.0 algorithm, to identify CNAs with a statistically significant frequency. A total of 20 significant gained regions were located on CFA 9, 10, 13, and 30. Those regions and the corresponding genes (reported in Table S3 in Supplementary Materials) were mostly overlapping with those showing higher penetrance across samples. The regions' size ranged from 33.9 Kb to 52.3 Mb, with a mean length of 4.2 Mb. CFA 10 and CFA 30 were affected by significant amplification in 36.8% (7/19) and 26.3% (5/19) of the samples, respectively. The three most frequent minimum common regions (MCRs) of CFA 10 were 1.7 to 1.9 Mb, 10.9 to 11.8 Mb and 43.6 to 45 Mb, while the most frequent MCRs of CFA 30 were 13.6 to 13.9 Mb, and 16.2 to 17.9 Mb. The GISTIC2.0 algorithm failed to identify statistically significant lost regions. A hierarchical clustering technique aimed to identify molecular features potentially correlated with the Ki67 index showed inconsistent results.

### Pathway Enrichment Analysis

**Figure 2:**
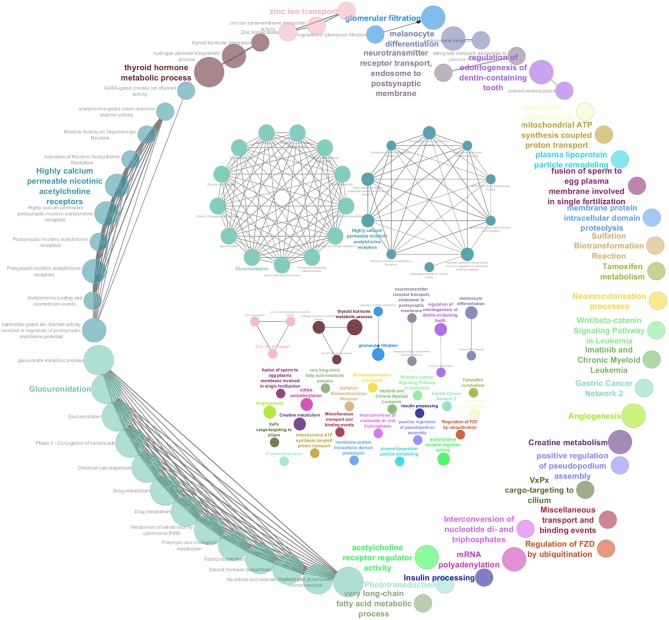
Graphic representation of the results obtained from the pathway enrichment analysis. These pathways were obtained subjecting the genes extrapolated from gained regions with a penetrance ≥25% (GR25 list) to ClueGO. They are represented all together in a circular shape as functional groups, which are visible in more detail inside the circle.

To generate a summary of the pathways likely involved in the tumorigenesis of COMs, four separate lists (i.e., GR25, GR40, LR25, and GS, see Methods) were submitted to the ClueGO tool to identify significantly enriched pathways. Pathways were considered significant if having an adjusted Benjamini-Hochberg *P* < 0.05. The enrichment analysis identified 60 significant pathways for the group GR25 (Figure 2), 10 significant pathways for the group LR25, and 49 significant pathways for the group GS. No pathways were found significantly enriched when analyzing the GR40 group. The complete list of significant pathways and genes is reported in Table S4 in Supplementary Materials, while the most interesting, together with corresponding genes, are listed in Table 1. Pathways found enriched in the GR25 group included *Angiogenesis* (*P* < 0.01), *Glucuronidation* (*P* < 0.01), *Highly calcium permeable nicotinic acetylcholine receptors* (*P* < 0.01), and *Wnt/beta-catenin Signaling Pathway in Leukemia* (*P* = 0.03). Interestingly, many pathways related to *Drug metabolism* (*P* < 0.01) were also found significantly enriched (see Table 1). Other significant pathways were *Gastric Cancer Network 2* (*P* = 0.01), *Chemical carcinogenesis* (*P* < 0.01), and *Insulin processing* (*P* = 0.01). Most of the other pathways found to be enriched were melanocytes-related, such as *Melanocyte differentiation* (*P* < 0.01), or linked to the dental apparatus, e.g., *Regulation of odontogenesis of dentin-containing tooth* (*P* < 0.01). Noteworthy, in the GR25 group and GS group, enriched pathways were mostly overlapping. Additionally, GS enriched pathways included the *T-helper 1 type immune response* (*P* < 0.01) and *Hippo-Yap signaling* pathways (*P* = 0.01). Regarding the LR25 group, the most enriched pathways were *Negative regulation of cell migration involved in sprouting angiogenesis* (*P* < 0.01), and *Hydrolysis of LPC* (*P* < 0.01).

**Table 1:** List of part of the significantly enriched pathways.

**Pathways**	**cPValue**	**Associated genes found**	**Source**
Angiogenesis	<0.01	ANGPT1, KDR, PDGFRA, PTK2	GR25, GS
Glucuronidation	<0.01	UGT2A1, UGT2A3, UGT2B10, UGT2B11, UGT2B15, UGT2B17, UGT2B28, UGT2B4, UGT2B7	GR25, GS
Highly calcium permeable nicotinic acetylcholine receptors	<0.01	CHRNA3, CHRNA5, CHRNB4	GR25, GS
Wnt/beta-catenin Signaling Pathway in Leukemia	0.03	FZD6, MYC, PYGO1, WIF1	GR25, GS
Drug metabolism	<0.01	DPYS, DUT, MGST3, RRM2B, UGT2A1, UGT2A3, UGT2B10, UGT2B11, UGT2B15, UGT2B17, UGT2B28, UGT2B4, UGT2B7	GR25, GS
Tamoxifen metabolism	0.02	SULT1E1, UGT2B15, UGT2B7	GR25, GS
Imatinib and Chronic Myeloid Leukemia	0.02	KIT, MYC, PDGFRA	GR25, GS
Gastric Cancer Network 2	0.01	ATAD2, DSCC1, FAM91A1, MYC	GR25, GS
Chemical carcinogenesis	<0.01	MGST3, UGT2A1, UGT2A3, UGT2B10, UGT2B11, UGT2B15, UGT2B17, UGT2B28, UGT2B4, UGT2B7	GR25, GS
Regulation of odontogenesis of dentin-containing tooth	<0.01	AMTN, ENAM, RSPO2, TNFRSF11B	GR25, GS
Insulin processing	0.01	EXOC1, MYO5A, RAB27A, SLC30A8	GR25
Melanocyte differentiation	<0.01	BLOC1S6, KIT, MYO5A, RAB27A, SLC24A5	GR25
T-helper 1 type immune response	<0.01	IL18R1, IL18RAP, IL1RL1, SOCS5, TRAPPC9, UTP3	GS
Hippo-Yap signaling	0.01	MAP4K4, NDRG1, STK3	GS
Negative regulation of cell migration involved in sprouting angiogenesis	<0.01	DLL4, SPRED1, THBS1	LR25
Hydrolysis of LPC	<0.01	JMJD7-PLA2G4B, PLA2G4B, PLA2G4D, PLA2G4E	LR25

*cPValue, P-value corrected with Benjamini-Hochberg; GR25, gains with penetrance ≥25%; LR25, losses with penetrance ≥25%; GS, regions highlighted as significant by Gistic analysis*.

### Comparative Analysis Between Canine and Human CNAs

Orthologous chromosomal regions in canine and human genome were examined to assess conserved CNAs between COMs and hMMs. In human literature, frequent gains of regions of human chromosomes HSA 1, 4, 5, 6, 7, 8, 11, 12, 17, 20, and losses of regions of HSA 3, 4, 6, 8, 9, 10, 11, 17, 21, have been reported (5, 7). The comparative analysis revealed 32 regions shared between COMs and hMMs, and regarded gains on CFA 9, 10, and 13, orthologous to HSA 17, 12, and 8–4 (respectively), and losses on CFA 11, 22 and 30, orthologous to HSA 9, 13, and 15 (respectively). A representation is visible in Tables 2A–C, while more details about the syntenic human regions are given in Table 3. In particular, the region characterized by the greatest recurrence was CFA 13:1722286–32543593, corresponding to HSA 8:99748261–136957380, already reported (5, 8, 24), and detected with a penetrance of 35% in this work. Regarding genes proposed as candidates for the tumorigenesis by other studies, a further concordance between COMs and hMMs was observed with the detection from gained regions of MYC, KIT and, for the first time, PDGFRA, although the latter was found gained only in the 12.5% of the hMMs analyzed (6) (Table 4A). A concordance can also be found in the loss of regions coding for BUB1B, KNSTRN, CYSLTR2, and SPRED1 (8) (Table 4B). Interestingly, also B2M was found imbalanced in both COMs and hMMs (8), but affected by a gain in the present work, instead of a loss. Other frequently reported events in hMMs are usually gains of BRAF (6, 8, 24), MDM2 (6, 8), CDK4 (5–7, 24), and CCND1 (5, 6, 24); losses of CDKN2A, PTEN (5, 6, 24), and TP53 (4, 6). However, aberrations involving the latter candidate genes were not found in the present study. A new promising target gene was also absent: PTPRJ was found lost in both hMMs and COMs (8), and was reported as inactivated by somatic mutations in COMs (28), but was not involved in the present cohort.

**Table 2:** Representation of the aberrated regions detected in the present study (CanFam3.1 annotation), showing a correspondent canine or human syntenic region in other studies, which are indicated through the bibliographic number: Wong et al. (8), Poorman et al. (24), Giannuzzi et al. (27), Hendricks et al. (28), Hayward et al. (6), Curtin et al. (5), Lyu et al. (7), and Furney et al. (4). CNAs are listed on the base of the chromosomal location, and divided into 3 groups: **(A)** gained regions with a penetrance ≥25% (GR25); **(B)** lost regions with a penetrance ≥25% (LR25); **(C)** represents gained regions found statistically significant by Gistic analysis (GS).

		**CANINE studies**				**HUMAN studies**					
**CHR**	**Present study**	**Wong et al. (8)**	**Poorman et al. (24)**	**Giannuzzi et al. (27)**	**Hendricks et al. (28)**	**Wong et al. (8)**	**Poorman et al. (24)**	**Hayward et al. (6)**	**Curtin et al. (5)**	**Lyu et al. (7)**	**Furney et al. (4)**
**(A) GR25**											
10	7814333–7827061					V					
	7827061–7896552					V					
	7896552–8860169					V					
	8860169–8889136					V					
	8889136–9197582					V					
	9225870–9593296					V					
	9593296–9724425					V					
	9724425–9752955					V					
13	1722286–32543593				V	V	V		V		
	32543593–34917864					V			V		
	34917864–60030824^*^					V			V		
							V		V		
	60030824–60110727						V				
30	16069762:16221290		V		V						
	16221290:16599297		V		V						
	16599298:16724522		V								
	16724522:16973676		V								
	16973676:17914975		V								
**(B) LR25**											
11	38095457–38199500								V		
	38199500–38219624								V		
	38219624–38219683								V		
	38219683–38263483								V		
	38263483–38372087								V		
22	102563–10473893						V				
30	1305764–2530849					V					
	2530849–2552538					V					
	2552538–2770691					V					
	2770691–4634866					V					
	4634866–5314049					V					
	5314049–9109654					V					
**(C) GS**											
9	1839468–1890597								V		
	2038907–2071775								V		
10	6204314–9752896					V					
	9948113–10047999					V					
13	101–23829651				V	V			V		
	23906345–60168042^*^					V	V		V		
									V		

Canine regions marked with ^*^ were syntenic to human regions belonging to two different chromosomes, and were split accordingly (view Table 3 for details).

**Table 3:** Comparison between canine CNAs detected in the present study (on the left, CanFam3.1 annotation), and corresponding syntenic human CNAs reported in literature (on the right, GRCh38/hg38 annotation).

**Shared canine-human regions**					
**Canine gains (present study)**	**Human gains**	**Canine loss (present study)**	**Human loss**	**Canine gistic (present study)**	**Human gains**
chr10:7814333–7827061	chr12:65226703–65241894^†^	chr11:38095457–38199500	chr9:18137869–18249633^§^	chr9:1839468–1890597	chr17:79737002–79797355^§^
chr10:7827061–7896552	chr12:65242092–65311784^†^	chr11:38199500–38219624	chr9:18249633–18271087^§^	chr9:2038907–2071775	chr17:79475861–79519528^§^
chr10:7896552–8860169	chr12:65311784–66424954^†^	chr11:38219624–38219683	chr9:18271087–18271147^§^	chr10:6204314–9752896	chr12:63095367–67388213^†^
chr10:8860169–8889136	chr12:66428185–66458897^†^	chr11:38219683–38263483	chr9:18271147–18348934^§^	chr10:9948113–10047999	chr12:67602904–67721312^†^
chr10:8889136–9197582	chr12:66458897–66810207^†^	chr11:38263483–38372087	chr9:18348934–18473616^§^	chr13:101–23829651	chr8:97763559–126022421^†§^
chr10:9225870–9593296	chr12:66840570–67211059^†^	chr22:102563–10473893	chr13:40898353–52112457^‡^	chr13:23906345–60168042	chr8:100M−140M^†‡§^
chr10:9593296–9724425	chr12:67213347–67350366^†^	chr30:1305764–2530849	chr15:32610232–33724875^†^		chr4:38M−65M^§^
chr10:9724425–9752955	chr12:67353025–67388264^†^	chr30:2530849–2552538	chr15:34810295–34839288^†^		
chr13:1722286–32543593	chr8:99748261–136957380^†‡§^	chr30:2552538–2770691	chr15:34839288–35104379^†^		
chr13:32543593-34917864	chr8:136957380-140179206^†§^	chr30:2770691-4634866	chr15:35104379-37107511^†^		
chr13:34917864–60030824	chr8:139727725–145066685^†§^	chr30:4634866–5314049	chr15:37107511–37907031^†^		
	chr4:41359607–70807315^‡§^	chr30:5314049–9109654	chr15:37907031–42087634^†^		
chr13:60030824–60110727	chr4:70738662–70861730^‡^				

*With ^†^the syntenic regions reported also by Wong et al. (8); with ^‡^the syntenic regions reported also by Poorman et al. (24); with an ^§^the syntenic regions reported by Curtin et al. (5); no regions were shared with Lyu et al. (7)*.

**Table 4:** Comparison of the target genes found gained **(A)** or lost **(B)** in this and other studies, which are indicated through the bibliographic number: Wong et al. (8), Poorman et al. (24), Giannuzzi et al. (27), Hendricks et al. (28), Hayward et al. (6), Curtin et al. (5), Lyu et al. (7), and Furney et al. (4).

	**Present study**	**CANINE studies**				**HUMAN studies**					
		**Wong et al. (8)**	**Poorman et al. (24)**	**Giannuzzi et al. (27)**	**Hendricks et al. (28)**	**Wong et al. (8)**	**Poorman et al. (24)**	**Hayward et al. (6)**	**Curtin et al. (5)**	**Lyu et al. (7)**	**Furney et al. (4)**
**(A) GAINED GENES**											
MYC	V	V	V			V	V				
KIT	V		V		V		V				
PDGFRA	V							V			
B2M^*^	V	V				V					
BRAF						V	V	V			
MDM2		V		V	V	V		V			
CDK4		V			V		V	V	V	V	
CCND1^*^		V	V			V	V	V	V		
TRPM7	V		V	V							
WIF1	V	V									
SLC27A2	V			V							
GABPB1	V			V							
USP8	V			V							
SPPL2A	V			V							
CYP19A1	V			V							
NOTCH1						V					
SMO		V				V					
TERT										V	
**(B) LOST GENES**											
BUB1B	V	V				V					
KNSTRN	V	V				V					
CYSLTR2	V	V				V					
SPRED1	V		V	V		V					
CDKN2A			V	V	V		V	V	V		
PTEN			V				V	V	V		
TP53								V			V
RB1	V	V	V								
LCP1	V	V									
FAM98B	V			V							
PTPRJ		V				V					
ARID1B						V					

With ^*^ the genes B2M and CCND1: B2M was found gained in the present study, but was reported as lost by Wong et al. (8); CCND1 was reported as gained in hMMs, but lost in COMs, by Wong et al. (8) and Poorman et al. (24).

### Comparison With Published Canine CNAs

Recently, several studies focused on COM's genetic landscape. The most frequently reported aberrations are gains on CFA 13, 17, and losses on CFA 11, 15, 22 (8, 24, 27, 28) together with a sigmoidal trend (a complex alternation of copy number gains followed immediately by copy number losses), in CFA 10 and 30 (8, 24, 28). In this study, CFA 10 and CFA 30 also showed the sigmoidal trend, and 7 of the regions found imbalanced were already reported in recent literature (Tables 2A,C). Among them, CFA 30:16069762:16221290 and 30:16221290:16599297, in particular, are reported in 2 other studies (24, 28), and had a penetrance ≥30% and ≥25% in this work, respectively.

Several genes which have been indicated to play a significant role in the development of COMs and hMMs were involved in CNAs, comprising gains of TRPM7 (24, 27), MYC (8, 24), KIT (24, 28), WIF1 (8), SLC27A2, GABPB1, USP8, SPPL2A, CYP19A1 (27) (Table 4A) and losses of RB1 (8, 24), LCP1, BUB1B, KNSTRN, CYSLTR2 (8), SPRED1 (24, 27), and FAM98B (27) (Table 4B). On the contrary, other genes such as MDM2 (27, 28) or CDK4 (8, 28), were not confirmed by our study. Finally, it is noteworthy that other genes involved in CNAs such as ADAM10, and genes belonging to gene families SNORA, SNORD, SLC25A, RPL, and RBM, have been recently shown to be highly expressed in metastatic COMs (32).

More details about the target genes taken into consideration (from both canine and human studies) are graphically represented in Tables 4A,B.

## Discussion

In the present study, more than 250 FFPE samples have been collected from archive material. However, due to the stringent inclusion criteria aimed to analyze only samples with a sufficient amount of paired healthy DNA, only 40 were considered adequate candidates for the aCGH analysis. The low number of samples has been a limitation, and a likely cause of the inconsistent results obtained from the hierarchical clustering, with only 5/20 cases with a Ki67 value <19.5. Since the presence of healthy tissue was a major restriction in the recruitment of cases, to ease future analysis the inclusion of a portion of presumed healthy tissue in the diagnostic sample is therefore recommended. The highly homogeneous cohort of samples obtained and the matching DNA for each sample allowed to overcome all potential discrepancies deriving from the use of genomic dog pools, which could have led to false correlations with race, age, sex, and health conditions.

A 50% increase in the DNA extraction yield was obtained by using the heptane as deparaffinization agent instead of the more toxic xylene (33). Precipitation of the extracted DNA with ethanol allowed to obtain a good DNA quality in some of the samples with an initial poor A^260^/A^230^ ratio.

Chromosomal aberrations detected in this study partially overlap with those already documented in other works on canine species, and the software analysis showed both new and known enriched pathways. Only the GR40 group failed to identify significantly enriched pathways, probably due to the limited number of genes included in the list.

The most characteristic aberration is confirmed to be the sigmoidal pattern of CFA 10 and CFA30 (8, 24, 28). However, the biological significance of these recurrently lost-gained regions is still unclear. Future studies correlating the presence of lost-gained regions and clinical data could improve our understanding of this specific molecular feature of COM.

MDM2 (8, 27, 28), CDK4 (8, 28), and CDKN2A (24, 27, 28), which were found altered by other authors, were not identified as significantly aberrated by GISTIC algorithm in this study. As reported also in hMM (4), MDM2 is known to favor tumor formation by acting on the tumor suppressor gene p53 (34). Although the reason for this discrepancy is not known, a significant gain of the MDM2 binding protein (MTBP) was instead present in our samples. These data, together with a copy number gain involving the p53 binding protein (TP53BP1), confirm the dysregulation of p53 family members in COMs. Moreover, a significant enrichment of the Hippo-Yap signaling pathway, which is strongly and sometimes contradictorily intertwined with the p53 pathway (35), was also detected.

CDK4 and its inhibitor CDKN2A are main actors of the cell cycle proliferation since they regulate Cyclin D1, allowing or not the transition from G1 to S phase. Although a direct involvement of CCND1 has been identified only in human acral and mucosal melanomas (5), and not in COMs (24), high expression of Cyclin D1 protein in COMs has been recently documented (36). Furthermore, a gain of the coding gene for Cyclin B2 (CCNB2), and a copy number imbalance of Eukaryotic Translation Initiation Factor family members (EIF2C2, EIF2AK4, EIF3E, EIF3H, EIF3J), were detected. Interestingly, the member EIF4E of the family is reported to increase the level of Cyclin D1 protein *in vitro* (37, 38), while other members (components of the EIF3 complex, in particular), have been correlated to human cancer (39–49), and human melanoma (41, 50, 51).

Another notable aberration is the loss of the tumor suppressor gene Retinoblastoma 1 (RB1), considered the governor of the cell cycle. RB1 has been already associated with other human and canine cancer types and it is strictly correlated to the cyclins' family. Even if with a mechanism different from those proposed in other works, cyclins activation appears then highly involved also in our cohort of COMs.

Among the genes related to cell proliferation and mitosis, GRHL2, a transcription factor able to bind the promoter region of TERT, was found to be gained. Although TERT is one of the most frequently involved genes in human non UV-induced melanomas (7, 10) it has never been found amplified in COMs. However, the gain of GRHL2 suggests that TERT expression may play a role in COMs.

A loss of mitosis-related genes was also highlighted. Loss of KNSTRN, required for correct chromosome segregation, BUB1, a checkpoint for mitosis progression, and TACC3, a stabilizer of the mitotic spindle, were detected.

COM is known to share with hMM the activation of MAPK and PI3K pathways, showing also similar responses to targeted therapies (52, 53). Aberrations are recognized to be part of the activation mechanism, and many MAPK-related genes are encoded by CFA 30 (orthologous to HSA 15) (8, 24, 28). Genes responsible for MAPK and PI3K activation found in this work comprise RASGRP1, MYC, FGF7, ANGPT1, TRPM7 (gained), and SPRED1 (lost). As reported in other works (6, 24), an imbalance of a wide variety of genes coding for tyrosine kinases receptors was also detected. These genes are abnormally activated in a wide range of human and animal tumors, inducing uncontrolled tumor proliferation (54). Our data showed an imbalance of the proto-oncogene c-KIT, and of other kinases such as PTK2, STK3, TEC, PDGFRA, VEGFR2, and CD63. It is noteworthy that VEGF receptors are involved in metastatic behavior in human melanoma studies (55), while PDGF receptors' expression has been shown to bear prognostic significance in COMs (22). Finally, CD63, a well-known melanoma-associated antigen (also called “Melanoma-Associated Antigen ME491”), has a role in VEGFA signaling.

As reported by other authors (28), the involvement of PI3K/mTOR/AKT signaling pathway is also suggested by the significant enrichment of the *Insulin processing* pathway, which is well-known to activate PI3K. The latter is also regulated by DEPTOR and MAP2K1 genes, which have been found altered in our study. Noteworthy, PI3K and mTOR are essential for the maintenance of the stem cell status in neural progenitor cells (56), and their abnormal involvement appears strategical for the tumorigenesis of neural crest-derived tumors such as COM.

Interestingly, the *Angiogenesis* pathway was enriched in both GR25 and GS lists (derived from the gained regions), and its suppressive pathway (*Negative regulation of cell migration involved in sprouting angiogenesis*) was enriched in LR25 list (derived from the lost regions). In addition, other vascular-related pathways, such as *Neovascularization processes*, were found enriched. Vessels proliferation, which plays an important role in several types of human melanomas (57), appears then to be deeply implicated in COMs' pathogenesis, and likely in their metastatic behavior. Neoangiogenesis is an obligatory phenotypic step for the establishment of distant metastases throughout the body: without an angiogenic process, the freshly-proliferated cells would incur into hypoxia and lack of nutrients, making the microenvironment adverse for the metastases survival (57). The main genes involved in endothelial and vascular cell proliferation, and reported by the ClueGo analysis, were ANGPT1, VEGFR2, PDGFRA, PTK2 for both GR25 and GS groups, and DLL4, SPRED1, THBS1, which are involved in the negative regulation of vessel sprouting, for LR25. Additionally, an imbalance (gains and losses) on the endothelin receptor type B gene (EDNRB), which role is crucial for melanocytes development (3) and for vessel homeostasis, was also detected. Interestingly, antibody-drug conjugates (ADC) targeting the endothelin B receptor (ET_B_R) both *in vitro* and *in vivo* systems (58, 59), show anti-tumor activity (even if partial) in hMMs (59). Given these premises, further evaluation of ET_B_R's expression in COMs could be promising to evaluate the application of ADC therapies also in dogs.

Furthermore, our data show the involvement of many other genes closely related to an angiogenic phenotype. Many genes coding for interleukin receptors, inflammatory molecules that contribute to neovascularization through endothelial cells migration and proliferation, and through metalloproteinases overexpression (as MMP19), were found gained in this study. Significantly, in hMM, a close relationship between IL-8, its receptor CXCR2, and MMP-2, is well-established (60, 61). Additional involved genes were those coding for fibroblast growth factors (FGF7 and FGF14), for lipid lysophosphatidic acids (LPAR6)—which are known to contribute to angiogenesis and lymphangiogenesis (57)—and for melanoma proliferation- and migration-related properties such as ZIC5 (62).

The GISTIC2.0 algorithm indicated the *T-helper 1 type immune response* pathway was significantly enriched (genes are listed in Table 1). Although in dogs therapeutic trials based on immunomodulation did not reach consistent results (63–65), these data strengthen the view of melanoma as a promising target for immunogenic therapies (66).

A general loss of many components of the T-cell homeostasis such as LCP1, TNFSF11, LRCH1, TRIM13 (which acts together with MDM2), RASGRP1, and GPR18 was found. On the contrary, the gain of PDCD7, involved in glucocorticoid-induced apoptosis in mouse T-cells (67), was detected.

Pathways related to drug metabolism were also found to be enriched, with *Glucuronidation* being the most significant. The cause is attributable to the high representation of the UGT-family genes, which are involved in the activity of the enzyme glucuronosyltransferase and phase II metabolism, and constitute an important pathway for xenobiotic elimination from the organism. Although the involvement of *Glucuronidation* in COM's behavior is still unclear, its potential role should be taken into consideration for future clinical trials and drug testing.

Not surprisingly, genes involved in the pathogenesis of other tumors were found to be altered. Significant gains of genes ATAD2, DSCC1, FAM91A1, and MYC brought to the enrichment of the *Gastric Cancer Network 2* pathway, and ATAD2 is a cancer-associated protein which can also induce the expression of Cyclin D1 and MYC (68).

Another enriched pathway, which has been frequently associated with cancer, was the *Wnt/beta-catenin Signaling Pathway in Leukemia*, which mediates the cell transduction signal. Gains of genes such as FZD6, PYGO1, and WIF1, causing Wnt inhibition, and MYC, were found. The involvement of Wnt signaling has been associated to numerous types of cancer, as glioblastoma (69), esophageal (70), ovarian (71), breast (72), colorectal (73), prostate (74), and lung (75) cancers and also to cutaneous melanomas (76). Gains of Wnt inhibitory genes in COM may still have a role in the complex deregulation of the Wnt pathway, which leads to carcinogenesis. However, further studies are needed to clarify this point.

The enrichment of pathways related to melanocytes' development (from neural stem cells) and pigmentation have been already reported in hMMs (7). In accordance, a significant enrichment of the *Highly calcium permeable nicotinic acetylcholine receptors*, and of the *Melanocyte differentiation* pathways were found in our study. Finally, the *Regulation of odontogenesis of dentin-containing tooth* pathway was also significantly enriched. Genes involved in the enrichment of this last pathway, namely AMTN, ENAM, RSPO2, and TNFRSF11B, encode, respectively, for the ameloblast protein amelotin, teeth component enamelin (a Wnt activator), and a TNF receptor. The involvement of these genes may be related to the frequent tendency of COMs to affect the oral cavity, and gingiva in particular (77). The application of aCGH on 19 COMs contributed to increase our knowledge of genetic aberrations in this canine tumor. We confirmed aberrational patterns noted also by other authors, as the sigmoidal trend in CFA 10 and 30. Thirty-two regions here detected showed to be syntenic with hMM-related regions and confirmed a common involvement of MAPK and PI3K pathways in COMs and hMMs. Moreover, our data suggest a strong involvement in COM's tumorigenesis of neovascularization-related pathways. These new data, together with the encouraging evidence of anti-angiogenic factors target therapies in human melanomas, remark the role of the dog as a model for hMMs and encourage new studies aimed to test the application of anti-angiogenic factors in the treatment of advanced and/or metastatic COMs.

## Data Availability Statement

The datasets generated for this study can be found in the NCBI's Gene Expression Omnibus, accession number: GSE131923.

## Ethics Statement

Ethical review and approval was not required for the animal study because only formalin-fixed, paraffin-embedded surgical biopsies from canine tumors were used in the present work. Tissues were provided by the archives of the Department of Comparative Biomedicine and Food Science (University of Padova) and of the Department of Animal Medicine and Surgery (Complutense University). Written informed consent for participation was not obtained from the owners because the biopsies collected from the archives had already been approved for research purposes by the attending veterinarians, at the moment they sent the material to our facilities.

## Author Contributions

SeF and MC conceptualized the study design. MC acquired the funds and supervised the whole project. CZ and EM-M collected and recruited the cases and their available information. GB and CZ performed the IHC analysis when necessary. SiF, MG, and MC reviewed H&E and IHC slides to confirm the diagnosis. GB and SeF performed the aCGH analysis. GB curated the processed data and wrote the original draft. All authors reviewed and approved the final manuscript.

### Conflict of Interest

The authors declare that the research was conducted in the absence of any commercial or financial relationships that could be construed as a potential conflict of interest.
