# Supplementary material for: Array Comparative Genomic Hybridization Analysis Reveals Significantly Enriched Pathways in Canine Oral Melanoma
Source: Front Oncol. 2019 Dec 12;9:1397. doi: 10.3389/fonc.2019.01397 (PMC6920211; doi:10.3389/fonc.2019.01397)
Supplement: Supplementary file 1 [file Data_Sheet_1.zip › Supplementary Material/Supplementary Material.docx]

**Table S1.** Selected samples included in the study, together with available clinical and histological data. F: female; FN: neutered female; M: male; MN: neutered male; G: good prognosis; B: bad prognosis.

**Table S2.** CNAs with penetrance≥25% (CanFam2 annotation), and lists of genes from gain and loss regions with penetrance≥25%, and penetrance≥40%.

**Table S3.** Significant CNAs obtained from GISTIC2.0 (CanFam2 annotation), and list of the corresponding genes.

**Table S4.** ClueGO significant pathways and list of genes applied for their detection. Pathways are listed in ascending order depending on the corrected term Pvalue.
